# GBT-1118 Rescued Impaired Bone Formation but Failed to Rescue Material Properties in Humanized Sickle-Cell-Disease Murine Model

**DOI:** 10.3390/cells15131209

**Published:** 2026-07-03

**Authors:** Kai Clarke, Wei He, Nikhil Menon, Tannin Schmidt, Alix Deymier, Marja Hurley

**Affiliations:** 1 Division of Endocrinology and Metabolism, Department of Medicine, School of Medicine, UConn Health, Farmington, CT 06032, USA; 2 Department of Biomedical Engineering, School of Dental Medicine, UConn Health, Farmington, CT 06032, USA

**Keywords:** sickle cell disease, hemoglobin allosteric modifier, voxelotor analog GBT1118, mechanical testing, material testing, bone disease

## Abstract

**Highlights:**

**What are the main findings?**

- GBT-1118 treatment restored hematocrit levels in both male and female humanized sickle-cell-disease mice.
- Sickle cell disease altered femoral bone composition and reduced bone mechanical strength in female mice, while GBT-1118 failed to rescue these material and mechanical defects.

**What are the implications of the main findings?**

- Improvements in anemia and erythrocyte lifespan alone may not be sufficient to reverse bone fragility associated with sickle cell disease.
- Additional therapies targeting bone quality and mechanical integrity may be necessary to effectively treat sickle cell bone disease.

**Abstract:**

Sickle cell disease (SCD), the most common inherited blood disorder in the United States, affects approximately 100,000 individuals annually. A major complication of SCD is sickle cell bone disease, which results in substantial bone loss comparable to osteoporosis. Previous work showed that treatment with GBT1118, a sickle hemoglobin polymerization inhibitor, improved bone formation and reduced bone resorption in humanized SCD mice. However, its effects on bone material and mechanical properties were unknown. To address this, four-month-old control and SCD mice were fed vehicle or GBT1118 chow for two months. Hematocrit levels, significantly reduced in SCD mice of both sexes, were restored by GBT1118 treatment, confirming its efficacy in improving anemia. Raman spectroscopy revealed increased mineral-to-organic matrix ratios in SCD femurs of both sexes and elevated carbonate-to-phosphate ratios in males, none of which were altered by GBT1118. Mechanical testing showed decreases in ultimate stress and Young’s modulus in female SCD femurs, with no significant differences in males nor rescue by GBT1118. Thus, while GBT1118 improved hematological parameters, it failed to restore the impaired bone material properties observed in SCD mice, highlighting the need for additional therapeutic strategies to address bone fragility in sickle cell disease.

## 1. Introduction

Studies have demonstrated that 2-hydroxy-6-[(2S)-1-(pyridine-3-carbonyl)piperidin-2-yl]methoxy (GBT1118)-supplemented chow prevents characteristic hypoxia-induced erythrocyte membrane damage while increasing hemoglobin–oxygen affinity in murine models of sickle cell disease (SCD) [1,2]. By stabilizing oxygenated hemoglobin and reducing hemoglobin S polymerization, GBT1118 improves oxygen delivery and decreases tissue hypoxia, thereby targeting the primary pathophysiologic mechanism underlying SCD. We previously reported that treatment of 4-month-old humanized SCD mice with GBT1118-supplemented chow for two months significantly increased hematocrit levels and enhanced bone formation through reductions in bone tissue hypoxia, improved osteogenesis and osteoblast function, and decreased osteoclast-mediated bone resorption [3]. These findings suggest that pharmacologic modulation of hemoglobin polymerization has beneficial effects that extend beyond hematologic parameters and may improve skeletal health. However, while improvements in bone quantity and cellular activity have been demonstrated, it remains unclear whether these changes translate into improvements in the intrinsic material properties of bone that ultimately determine resistance to fracture. Therefore, the present study investigates the effects of long-term GBT1118 treatment on the material properties of femoral bone in both male and female humanized SCD mice.

Sickle cell disease is the most common inherited hemoglobinopathy in the United States, affecting approximately 100,000 individuals and millions worldwide [4]. The disease results from a single nucleotide substitution in the β-globin gene on chromosome 11p15, causing the replacement of glutamic acid with valine at the sixth amino acid position of the β-hemoglobin chain. This seemingly minor genetic alteration profoundly changes the biochemical properties of hemoglobin, promoting polymerization under deoxygenated conditions. The resulting rigid hemoglobin polymers distort erythrocytes into the characteristic sickle shape, reducing cellular deformability and increasing erythrocyte fragility. Sickled erythrocytes exhibit shortened lifespans, impaired oxygen transport, and an increased tendency to obstruct the microvasculature, leading to chronic hemolytic anemia, recurrent vaso-occlusive events, tissue hypoxia, and progressive multi-organ damage. These pathophysiologic processes underlie many of the debilitating clinical manifestations of SCD, including chronic pain, acute pain crises, stroke, pulmonary complications, renal dysfunction, and musculoskeletal disease.

Among the chronic complications of SCD, skeletal abnormalities are particularly prevalent and contribute substantially to morbidity and reduced quality of life. Sickle cell bone disease encompasses a spectrum of musculoskeletal manifestations, including osteopenia, osteoporosis, osteonecrosis, bone infarction, delayed fracture healing, and an increased incidence of fragility fractures. Patients frequently exhibit reduced bone mineral density despite standard supportive therapies, including chronic transfusion regimens [5]. The pathogenesis of sickle cell bone disease is multifactorial and involves chronic marrow hyperplasia secondary to anemia, repeated vaso-occlusive episodes, persistent tissue hypoxia, inflammation, oxidative stress, and dysregulated bone remodeling characterized by impaired osteoblast activity and excessive osteoclast-mediated bone resorption. Collectively, these mechanisms compromise both bone architecture and mechanical competence, increasing susceptibility to fracture.

Current therapeutic strategies for SCD primarily focus on reducing disease complications rather than correcting the underlying molecular defect of hemoglobin S polymerization. Conventional treatments include hydroxyurea therapy, chronic blood transfusions, and supportive care aimed at preventing vaso-occlusive crises and end-organ damage. Curative approaches, including allogeneic hematopoietic stem cell transplantation and, more recently, gene-editing and gene-addition therapies approved by the U.S. Food and Drug Administration, directly address the genetic basis of the disease [6,7]. However, these treatments remain inaccessible for many patients because of limited donor availability, procedural risks, specialized infrastructure requirements, and substantial financial costs. Consequently, pharmacologic therapies that target hemoglobin polymerization while remaining broadly accessible continue to represent an important area of investigation.

Although our previous work demonstrated that GBT1118 improves bone formation and suppresses bone resorption in humanized SCD mice, it remains unknown whether these biologic improvements result in stronger, mechanically superior bone. Bone mass and microarchitecture do not necessarily predict bone quality, as the intrinsic material properties of bone tissue—including stiffness, strength, and resistance to deformation—also contribute substantially to fracture risk. Therefore, the objective of the present study was to determine whether long-term treatment with GBT1118 improves the material properties of femoral bone in male and female humanized SCD mice. Despite the previously observed improvements in bone remodeling, our findings indicate that long-term GBT1118 treatment did not significantly improve the material properties of bone, suggesting that enhancements in bone formation alone may be insufficient to restore bone quality in SCD.

## 2. Materials and Methods

### 2.1. Experimental Mice

Male and female control (Ctrl) and humanized Townes sickle-cell-disease (SCD) mice on mixed C57BL/6;129 genetic backgrounds were purchased from The Jackson Laboratory (Stock number: 013071, Bar Harbor, MA, USA) and housed by genotype in the Center for Comparative Medicine at UConn Health. Townes mice harbor the human α- and β-hemoglobin genes which have been knocked into the mouse locus [8]. By crossing the heterozygous mice (sickle-cell-trait, SCT) one can generate homozygous (SCD) mice and healthy control littermates. To avoid confounding effects due to chronic kidney disease as described in other studies [9], mice were not aged beyond 6 months old. At 4 months old, healthy control mice were treated with vehicle chow (Teklad 2020 Diet, vehicle) consisting of 1% calcium, 0.7% total phosphate and 1.5 IU D3/g. Age- and sex-matched SCD mice were treated with either vehicle or GBT1118 chow (Teklad 2020 Diet with 4 g/kg GBT1118). Mice were sacrificed after 2 months of treatment. All animal protocols were approved by the UConn Health Institutional Animal Care and Use Committee.

### 2.2. Hematocrit Measurement

Whole blood was collected from the hearts of control and SCD mice. Blood was then placed in shortened capillary tubes which were thoroughly sealed. The sealed tubes were centrifuged at 12,000 RPM for 5 min. Immediately after being removed from the centrifuge, the tubes were then carefully placed on a micro-hematocrit reader, ensuring the top of the plasma layer intersected with the 100% line. The percentage corresponding to the height of the packed red blood cells was then read and recorded from the scale. Hematocrit was then estimated by calculating the ratio of the packed erythrocytes to the total length of the sample in the capillary tube [10].

### 2.3. Raman Spectroscopy Analysis

Excised femurs from 6-month-old control mice treated with either vehicle or GBT1118 chow were cleaned of all soft tissue, wrapped and stored in gauze dampened with Phosphate-Buffered Saline (PBS) prior to analysis. Samples were dried and mounted onto a clean microscope slide using dental wax. Raman spectroscopy was conducted using a WitecAlpha300 R-Confocal Raman microscope (Witec, Ulm, Germany). Samples were analyzed using a 785 nm laser with a laser power of ~40 mW, a 50× objective with a working distance of 9.1 mm, and a 30 s acquisition time. Per sample, five Raman spectra were acquired to reduce noise and background signal. Peaks of interest within each spectrum were fit using Lorentzian curves: 960 Δcm^−1^ phosphate peak, 1070 Δcm^−1^ carbonate peak and 1660 Δcm^−1^ Amide I peak. Peak areas were then used to calculate carbonate-to-phosphate ratios (1070/960), phosphate-to-Amide ratios (960/1660) and carbonate-to-Amide ratios (1070/1660).

### 2.4. Evaluation of Femur Bone Mechanics

Three-point bend tests were conducted on the same femurs subjected to Raman spectroscopy. The femurs were placed in the Biomomentum Mach-1 three-point bend system with the posterior face undergoing compressive loading using an 8 mm span. Femurs were loaded at a rate of 0.1 mm/s until failure using a 25 kg load cell. The second moment of inertia and average centroid of the bone were calculated from microCT data using the BoneJ plug-in in ImageJ version 2.14.0. MicroCT was conducted at an 8 mm^3^ resolution, 55 kVp, 145 mA, 600 ms integration time and 1000 projections/180°. Material properties were calculated from mechanical and structural properties using the Euler–Bernoulli engineering beam theory. Young’s modulus was determined from the slope of the stress–strain curve. The yield force was deemed the point where the stress–strain curve deviated from the linear region. Ultimate stress was the maximum stress measured during the test.

### 2.5. Statistical Analysis

GraphPad Prism 10 (Dotmatic, Boston, MA, USA) was used for statistical analysis, specifically to conduct one-way ANOVA tests on each dataset, followed by Tukey tests for post hoc multiple comparisons. Differences were considered significant when the *p* value was less than 0.05. Data are displayed as mean ± standard error of measurement (SEM) or graphically as mean with individual data points.

## 3. Results

### 3.1. GBT-1118 Rescues Decreased Hematocrit Levels in SCD Mice

**Figure 1:**
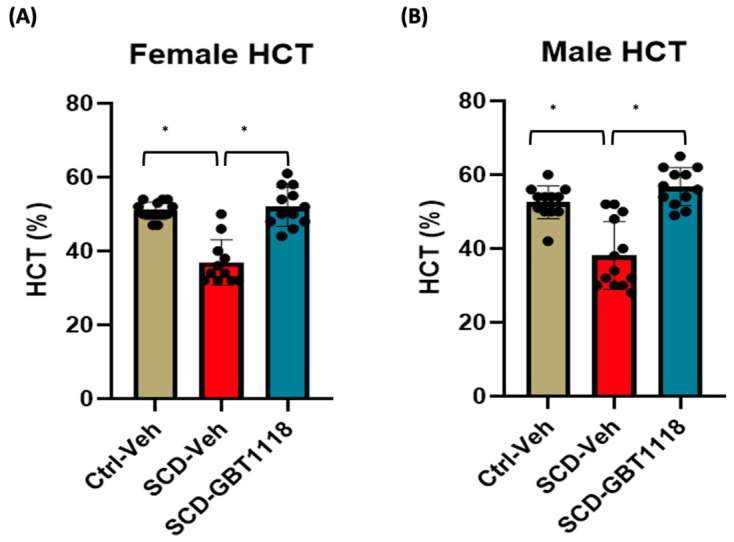
Measurement of hematocrit (HCT). HCT was measured in (**A**) 6-month-old control and SCD females treated with vehicle or GBT1118 chow and (**B**) 6-month-old male mice treated with vehicle or GBT1118 chow. Compared with control mice, HCT was significantly decreased in SCD mice of both sexes treated with vehicle chow but was significantly increased by GBT1118 treatment for 2 months. Data shown as bar graphs with mean, and SEM with individual data points. n = 11–14 mice/group. * *p* < 0.05 by one-way ANOVA.

A characteristic marker of SCD in human patients is a decrease in hematocrit levels caused by the decreased lifespan of sickle erythrocytes [11]. This characteristic decrease was seen in our study where 4-month-old SCD mice treated with vehicle chow for 2 months had significantly lower hematocrit levels compared to their healthy control littermates treated with vehicle chow. SCD mice treated for 2 months with GBT1118 chow exhibited a significant increase in hematocrit levels compared to vehicle-treated SCD mice. These changes were mirrored in both the male and female mice with no sexual dimorphism observed (Figure 1A,B).

### 3.2. Raman Spectroscopy Analysis

**Figure 2:**
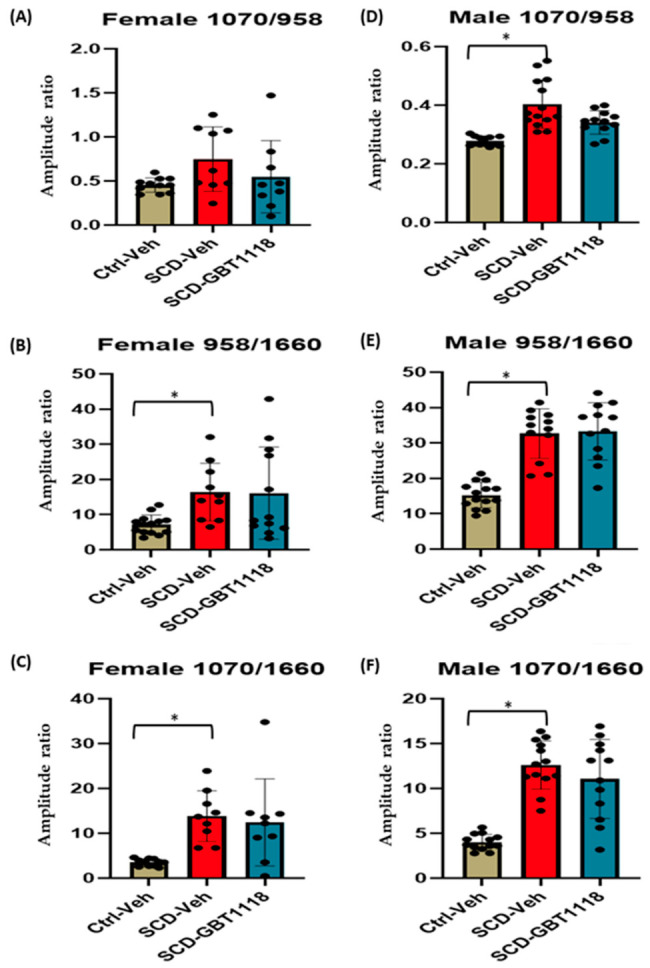
Raman analysis of compositional properties of femurs. (**A**) The carbonate-to-phosphate ratio was unchanged between vehicle-treated SCD female mice and control mice. (**B**) The phosphate-to-Amide I ratio and (**C**) carbonate-to-Amide I of SCD female mice treated with vehicle chow were significantly higher than that of the control female mice treated with vehicle chow. (**D**) The carbonate-to phosphate ratio, (**E**) phosphate-to-Amide I and (**F**) carbonate-to-Amide I were all significantly higher in vehicle-treated male SCD mice compared to controls. GBT1118 had no effect on male nor female ratios. Data shown as bar graphs with mean, and SEM with individual data points. n = 11–14 mice/group. * *p* < 0.05 by one-way ANOVA.

Femurs excised from 4-month-old control mice and SCD mice treated for 2 months with either vehicle chow or GBT1118 chow were subjected to Raman spectroscopy to probe the effect of sickle cell disease on the composition of the resultant bone, specifically in the diaphyseal cortical bone. The carbonate-to-phosphate ratio, which indicates the amount of carbonate ions substituted within the phosphate lattice, was significantly higher in the SCD male mice treated with vehicle chow compared to the control mice (Figure 2D). The carbonate-to-phosphate ratio, indicative of bone turnover, was unchanged in female SCD mice (Figure 2A). GBT1118 chow had no significant effect for either sex. The phosphate-to-Amide I ratio, which is a measure of the amount of mineral (phosphate) relative to the amount of organic matrix (collagen), was significantly higher in vehicle-treated SCD mice compared to the control mice (Figure 2B,E). Similarly, the carbonate-to-Amide I ratio, indicative of the amount of mineral (carbonate within hydroxyapatite) relative to the amount of organic matrix (collagen), was significantly higher in SCD mice treated with vehicle chow compared to vehicle-treated controls (Figure 2C,F). The trends seen in the mineral-to-organic matrix ratios were mirrored in both sexes, and GBT1118 chow treatment had no significant effect on either of the ratios.

### 3.3. Material Mechanical Properties

**Figure 3:**
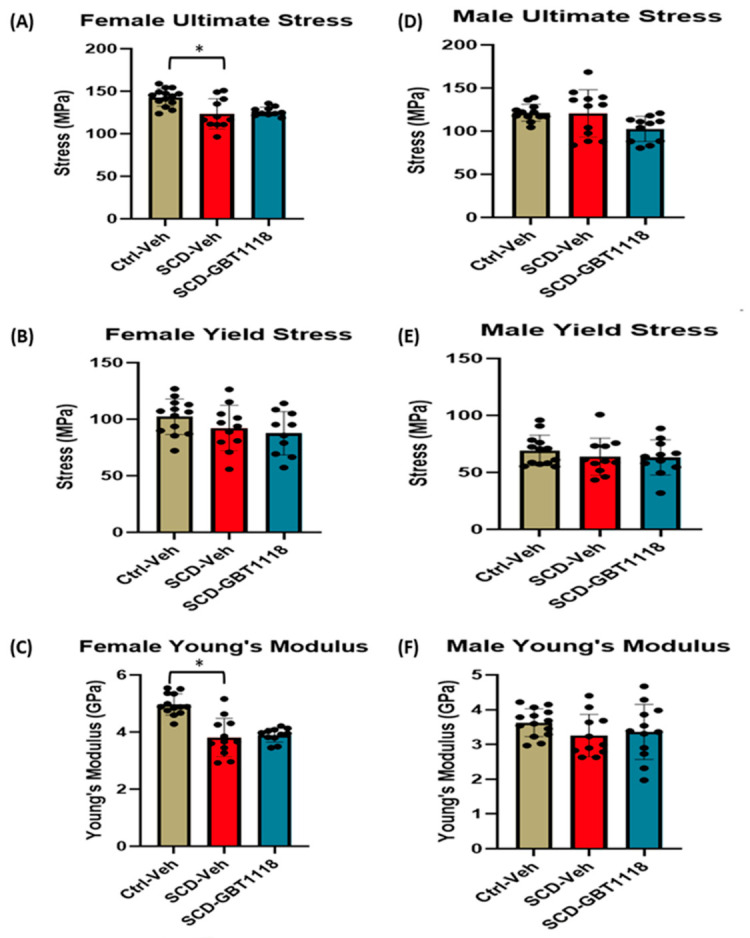
Assessment of mechanical properties of femurs. (**A**) The ultimate stress of vehicle-treated female SCD femurs was significantly decreased compared to controls. (**B**) The yield stress was unchanged in SCD female mice compared to controls while (**C**) the Young’s Modulus was significantly decreased in vehicle-treated SCD female mice compared to controls. (**D**) The ultimate stress, (**E**) yield stress and (**F**) Young’s modulus were all unchanged in vehicle-treated SCD male mice compared to controls. GBT1118 had no effect on mechanical properties in neither female nor male mice. Data shown as bar graphs with mean, and SEM with individual data points. n = 11–14 mice/group. * *p* < 0.05 by one-way ANOVA.

Excised femurs that were previously subjected to Raman analysis were then analyzed via a three-point bend test [12] to assess the mechanical properties of the femurs. Ultimate stress, yield stress, and Young’s modulus can be calculated from normalizing the mechanical testing data with structural properties derived from μCT. The ultimate stress, which indicates the maximum amount of stress a bone can withstand when under load before undergoing fracture, was significantly decreased in vehicle-treated SCD female mice compared to vehicle-treated controls (Figure 3A). The yield stress was unchanged between the groups in female femurs (Figure 3B). Female femurs also exhibited a lower Young’s modulus compared to the control vehicle-treated mice (Figure 3C). Regarding male femurs, there was evidence of sexual dimorphism as there was no significant difference seen among the groups regardless of genotype or treatment for any of the probed mechanical properties (Figure 3D–F).

## 4. Discussion

We previously determined the effect of GBT1118 treatment on the sickle cell bone disease phenotype in a humanized SCD murine model. We reported that in both sexes, SCD caused a significant decrease in bone formation and significantly increased bone resorption, both of which were rescued with GBT1118 treatment [7]. Despite these positive results, the effects of SCD and GBT1118 treatment on the bone structure as well as its compositional and mechanical properties remained unknown. The findings in this study suggest that restoration of erythrocyte lifespan alone may be insufficient to reverse skeletal deficits once they have become established. Bone remodeling occurs over a substantially longer timescale than hematologic recovery, and, therefore, structural abnormalities that accumulate during chronic disease progression, including increased cortical porosity and altered tissue mineralization, may persist despite improvements in systemic oxygen delivery. These findings are consistent with the concept that skeletal complications of SCD are multifactorial and arise not only from hemolytic anemia but also from chronic inflammation, oxidative stress, repeated vaso-occlusive episodes, and alterations in the bone marrow microenvironment. Consequently, therapies directed solely toward reducing hemoglobin polymerization may require combination with skeletal-targeted interventions to fully restore bone quality.

In this study we first confirmed whether GBT1118 chow could modulate hematocrit levels in male and female SCD mice compared to controls. A characteristic of SCD in human patients is a significant reduction in hematocrit levels, which indicate the amount of red blood cells present in your whole blood [11]. In SCD patients, red blood cells rapidly undergo decay due to their sickle shape, resulting in significantly lower hematocrit levels. This characteristic phenotype was observed in our male and female vehicle-treated SCD mice, which exhibited significantly lower hematocrit levels compared to controls. The administration of GBT1118 chow to SCD mice was able to significantly rescue the decreased hematocrit levels in both male and female mice, which implies that GBT1118 can significantly increase the lifespan of sickle erythrocytes in SCD mice.

The increased mineral-to-matrix ratios observed in vehicle-treated SCD mice also warrant further consideration. Although increased tissue mineralization is often associated with stronger bone, excessive secondary mineralization can adversely affect the mechanical behavior of bone tissue [12]. Bone is a composite material in which minerals provide stiffness while the collagen matrix contributes toughness and resistance to crack propagation. When remodeling is reduced or bone packets remain in the skeleton for prolonged periods, continued mineral deposition increases tissue stiffness but simultaneously decreases the ability of bone to absorb energy before failure [12]. Thus, hypermineralized bone may become increasingly brittle despite exhibiting greater mineral content. The elevated phosphate-to-Amide I and carbonate-to-Amide I ratios observed in the present study therefore likely reflect increased tissue age and altered remodeling dynamics rather than improved bone quality. This interpretation is further supported by our previous findings demonstrating impaired bone remodeling in the same animal model [3].

Though rarely reported, patients with SCD can also develop osteosclerosis [5], which has a characteristic increase in BMC compared to controls and would also coincide with our current Raman data. Studies have also highlighted that despite the increase in bone mineral content seen in these SCD patients, it was not enough to protect them from clinical fractures [2], implying that the bone is still quite weak. Although increased bone mineral content is generally associated with increased bone stiffness, excess mineralization reduces mineral toughness, increasing the risk of fractures [13,14]. Although male and female SCD mice exhibited similar increases in mineral-to-matrix ratios and comparable hematocrit rescue following GBT1118 treatment, female SCD mice demonstrated significantly greater cortical porosity than males [3]. Increased cortical porosity has been shown to substantially impair bone mechanical performance independent of tissue mineralization [15,16]. Consequently, similar Raman-derived compositional signatures may not necessarily translate into equivalent mechanical behavior when differences in bone microarchitecture are present.

The discrepancy between the compositional and mechanical findings further emphasizes the importance of evaluating bone quality using complementary techniques. Raman spectroscopy provides valuable insight into tissue composition but does not account for microarchitectural deterioration or structural defects that substantially influence whole-bone mechanics. Although both male and female SCD mice exhibited increased mineral-to-matrix ratios, only female mice demonstrated significant reductions in ultimate stress and Young’s modulus. These findings suggest that tissue composition alone is insufficient to predict mechanical competence. Instead, mechanical integrity likely reflects the combined effects of tissue composition, cortical porosity, microstructural organization, and collagen quality. Indeed, our previous microCT analysis demonstrated significantly increased cortical porosity in female SCD mice, which likely reduced the effective load-bearing cross-sectional area despite increased tissue mineralization. Together, these observations highlight the multifactorial nature of skeletal fragility in sickle cell disease.

Studies have reported that sex does not play a major role in the prevalence of sickle cell disease, with both males and females being affected equally [17,18]. Consequently, the greater susceptibility of female SCD mice to mechanical deterioration was an unexpected finding. However, sex-dependent differences in skeletal remodeling may partially explain these observations. Estrogen plays a central role in regulating cortical-bone remodeling and osteocyte survival [19], and chronic inflammatory conditions have been shown to differentially affect female bone metabolism [20]. Furthermore, prolonged marrow expansion and repeated vaso-occlusive injury may alter intracortical remodeling differently between sexes, potentially resulting in greater accumulation of structural defects in females despite comparable hematologic improvement following GBT1118 treatment. Although the current study was not designed to investigate the underlying molecular mechanisms responsible for these sex-specific responses, these findings emphasize the importance of considering sex as a biological variable when evaluating skeletal complications of sickle cell disease and assessing therapeutic efficacy.

In summary, the administration of GBT1118 chow proved to be beneficial in our previous study, and this trend was also seen in some of our current data. GBT1118 chow was able to significantly increase the lifespan of sickle erythrocytes in both sexes; however, its effect on the material and mechanical properties varied. This study demonstrated that correction of hematologic abnormalities alone is insufficient to restore normal bone quality in sickle cell disease. Although GBT1118 effectively improved hematocrit levels, these improvements did not translate into recovery of cortical bone composition or mechanical integrity. The persistence of altered tissue mineralization together with compromised mechanical properties, particularly in female mice, suggests that skeletal deterioration may become partially uncoupled from hematologic disease progression once structural damage has accumulated. These findings provide further evidence that preservation of bone quality in sickle cell disease will likely require therapeutic approaches that target both the underlying hematologic disorder and the skeletal remodeling abnormalities responsible for long-term bone fragility.

## Data Availability

The original contributions presented in this study are included in the article. Further inquiries can be directed to the corresponding authors.
